# Temperature Control of *psaA* Expression by PsaE and PsaF in Yersinia pestis

**DOI:** 10.1128/JB.00217-19

**Published:** 2019-07-24

**Authors:** Joshua D. Quinn, Eric H. Weening, Taryn A. Miner, Virginia L. Miller

**Affiliations:** a Department of Microbiology and Immunology, University of North Carolina, Chapel Hill, North Carolina, USA; b Department of Genetics, University of North Carolina, Chapel Hill, North Carolina, USA; Michigan State University

**Keywords:** PsaA, RNA thermometer, TcpP, ToxR, *Y. pestis*, pH, pH 6 antigen, temperature

## Abstract

PsaA, the subunit of the fimbria originally referred to as the “pH 6 antigen,” is required for full virulence of Yersinia pestis during bubonic plague. The expression of *psaA* is dependent upon specific environmental signals, and while the signals (high temperature and acidic pH) are defined, the mechanisms underlying this regulation remain unclear. In the closely related species Yersinia pseudotuberculosis, *psaA* transcription requires two regulatory genes, *psaE* and *psaF*, and it is speculated that posttranscriptional regulation of PsaE and/or PsaF contributes to the regulation of *psaA* transcription. Few studies have examined the regulation of *psaA* expression in Y. pestis, and prior to this work, the roles of *psaE* and *psaF* in Y. pestis had not been defined. The data presented here show that both *psaE* and *psaF* are required for *psaA* transcription in Y. pestis and that the impact of temperature and pH is mediated through discrete posttranscriptional effects on PsaE and PsaF. By generating antibodies that recognize endogenous PsaE and PsaF, we determined that the levels of both proteins are impacted by temperature and pH. High temperature is required for *psaE* and *psaF* translation via discrete mechanisms mediated by the mRNA 5′ untranslated region (UTR) upstream of each gene. Additionally, levels of PsaE and PsaF are impacted by pH. We show that PsaF enhances the stability of PsaE, and thus, both PsaE and PsaF are required for *psaA* transcription. Our data indicate that the environmental signals (temperature and pH) impact the expression of *psaA* by affecting the translation of *psaE* and *psaF* and the stability of PsaE and PsaF.

**IMPORTANCE** Y. pestis is a Gram-negative bacterial pathogen that causes bubonic plague. As a vector-borne pathogen, Y. pestis fluctuates between an arthropod vector (flea) and mammalian host. As such, Y. pestis must recognize environmental signals encountered within each host environment and respond by appropriately regulating gene expression. PsaA is a key Y. pestis mammalian virulence determinant that forms fimbriae. Our work provides evidence that Y. pestis utilizes multiple posttranscriptional mechanisms to regulate the levels of two PsaA regulatory proteins in response to both temperature and pH. This study offers insight into mechanisms that bacteria utilize to sense environmental cues and regulate the expression of determinants required for mammalian disease.

## Abstract (precis)

Y. pestis is a Gram-negative bacterial pathogen that causes bubonic plague. As a vector-borne pathogen, Y. pestis fluctuates between an arthropod vector (flea) and mammalian host. As such, Y. pestis must recognize environmental signals encountered within each host environment and respond by appropriately regulating gene expression. PsaA is a key Y. pestis mammalian virulence determinant that forms fimbriae. Our work provides evidence that Y. pestis utilizes multiple posttranscriptional mechanisms to regulate the levels of two PsaA regulatory proteins in response to both temperature and pH. This study offers insight into mechanisms that bacteria utilize to sense environmental cues and regulate the expression of determinants required for mammalian disease.

## INTRODUCTION

Yersinia pestis is a vector-borne bacterial pathogen that causes plague, a fulminant disease that manifests in multiple forms (bubonic, pneumonic, and septicemic) and is responsible for three major pandemics (1–3). Bubonic plague, the most common form of the disease in humans, occurs after Y. pestis is deposited by a flea into the dermal layer of skin and rapidly disseminates to distal tissue and into systemic circulation (4–9). Y. pestis is the only member of the Enterobacteriaceae family to rely on an arthropod-vector (flea) for transmission to a mammalian host, which occurs during a blood meal. The transmission of Y. pestis to a flea from an infected mammal also occurs during a blood meal and requires high levels of bacteria in mammalian blood (∼10^8^ CFU/ml); thus, both survival within a flea and systemic dissemination within a mammalian host are essential for the biphasic life cycle of Y. pestis (10). Transcriptome analyses reveal distinct Y. pestis expression profiles within a flea and mammalian host (11–13), suggesting that Y. pestis regulates gene expression in response to differential environmental signals encountered within each host. While the signals that distinguish the mammalian and flea microenvironments are not well defined, the temperature difference between a flea (∼26°C) and mammalian host (37°C) is thought to serve as a key environmental signal, as the expression of many Y. pestis virulence genes increases following an upshift in temperature from 26°C to 37°C (13, 14).

One such temperature-regulated virulence factor of Y. pestis is the “pH 6 antigen” (PsaA). PsaA forms fimbria-like structures on the cell surface (15, 16) and is required for the full virulence of Y. pestis in multiple mouse models of disease (15, 17–20). While the functional role of PsaA during mammalian infection has not been defined, *in vitro* studies using various cell lines suggest that PsaA functions to both inhibit phagocytosis and promote host cell adherence (21, 22). Intriguingly, PsaA production requires a combination of high temperature (>35°C) and acidic pH (pH <6.7) (23), and, since the detection of *psaA* transcripts corresponds with the detection of PsaA (24), it is predicted that Y. pestis utilizes temperature and pH to regulate the transcription of *psaA*. Despite this unusual expression pattern, the regulation of *psaA* transcription has not been examined in Y. pestis, and the mechanisms by which temperature and pH contribute to *psaA* transcription are not known.

The *psa* locus consists of five genes responsible for the production, translocation, and assembly of PsaA subunits into fimbria-like structures; *psaE* and *psaF* encode regulators, *psaA* encodes the fimbrial subunit, and *psaB* and *psaC* encode proteins that resemble the PapD and PapC families of chaperone and usher proteins, respectively (16). In the absence of *psaBC*, PsaA accumulates in the cell, indicating that the products of these genes contribute to PsaA export (15, 16). Encoded upstream of *psaA*, PsaE and PsaF are predicted to be transcriptional activators that coregulate *psaA* transcription (15, 24). However, the precise role(s) of PsaE and PsaF in Y. pestis is not thoroughly understood, and their predicted function(s) is extrapolated in part from studies in Yersinia pseudotuberculosis (*psa*) and of homologues in the closely related species Yersinia enterocolitica (*myf*) (25–27). In Y. pseudotuberculosis, *psaE* and *psaF* are both required to detect *psaA* transcripts (27). PsaE is predicted to have a DNA-binding domain, and there is evidence that PsaE contributes to *psaA* expression in Y. pestis (15, 24); however, PsaF has no conserved domains, and its role remains elusive. Analysis of fusions of alkaline phosphatase to PsaE and PsaF in Y. pseudotuberculosis suggests that both proteins are integral membrane proteins, each with a single transmembrane domain (27). While pairs of regulatory proteins with similar topology to PsaE and PsaF have been identified in other bacteria (28–31), PsaE and PsaF exhibit little primary sequence similarity with known proteins. The transcription of *psaE* and *psaF* in Y. pseudotuberculosis is not affected by temperature or pH, and thus, the transcription of these regulatory genes is not sufficient to activate *psaA* transcription (27). This has led to speculation that the production and/or activity of PsaE/F is subject to posttranscriptional regulation (27). However, it has yet to be determined whether PsaE and/or PsaF is influenced by temperature or pH and how PsaE and PsaF influence the expression of *psaA*.

We set out to define the mechanisms that contribute to the regulation of PsaA production in Y. pestis by PsaE and PsaF. In addition to monitoring *psaEF* expression, we generated antibodies against both PsaE and PsaF to monitor the levels of these proteins. Analysis of *psaE* and *psaF* transcription and PsaE and PsaF protein levels revealed that PsaE and PsaF are impacted by temperature and pH via posttranscriptional mechanisms. Our data suggest that temperature affects synthesis and that pH influences the stability of these key regulators of *psaA* expression.

## RESULTS

### Temperature and pH provide discrete signals that activate *psaA* transcription in Y. pestis.

**FIG 1:**
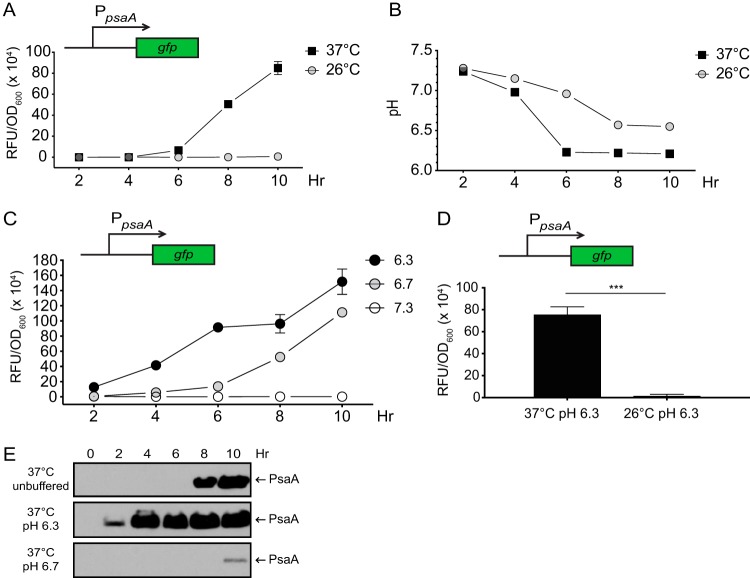
Expression of a *psaA* transcriptional reporter and production of PsaA require high temperature and low pH. WT Y. pestis (YP6) carrying a *psaA* transcriptional reporter (pEW102, *psaA-gfp*) was grown at 26°C and 37°C, and *psaA* transcription (A, C, and D), medium pH (B), and PsaA (E) were analyzed as described in Materials and Methods. (A and B) *psaA* expression (A) and growth medium pH (B) were determined following growth at 26°C and 37°C in unbuffered BHI broth over time. (C) WT carrying *psaA-gfp* was grown at 37°C in BHI broth buffered to pH 6.3, 6.7, and 7.3, and reporter expression was determined. (D) WT carrying *psaA-gfp* was grown for 8 h at 37°C and 26°C in buffered BHI broth, and reporter expression was determined. Each bar represents the mean RFU/OD_600_, and error bars represent standard deviations. For reporter experiments, each sample was assayed in biological triplicates. ***, *P* < 0.0001 using Student’s *t* test to compare mean values. (E) Whole-cell lysates of WT grown in BHI broth at 37°C were prepared, and PsaA was analyzed via Western blotting, as described in Materials and Methods. At least three independent experiments were performed. Data presented are from a representative experiment.

Temperature and pH are well-established environmental signals that affect the expression of *psaA* in Y. pestis (15, 23, 24). Despite this, the mechanisms utilized by Y. pestis to regulate the transcription of *psaA* in response to high temperature (>35°C) and mildly acidic pH (<6.7) are not known. To investigate how temperature and pH affected *psaA* expression and PsaA production in Y. pestis, a *psaA-gfp* transcriptional reporter (pEW102) was introduced into Y. pestis strain CO92 cured of the virulence plasmid pCD1 (YP6; here referred to as the wild-type strain [WT]). Prior studies on *psaA* expression used cultures grown under conditions in which the starting pH of the growth medium was adjusted but was not buffered to maintain the pH during growth (15, 24, 27). Thus, we first examined the expression of *psaA-gfp* in WT grown in unbuffered brain heart infusion (BHI) broth at 26°C and 37°C; both expression and the pH of the medium were monitored over time (Fig. 1A). Consistent with previous studies, *psaA* expression was observed only at 37°C. During growth at 37°C, *psaA-gfp* activity was initially detected at 6 h (late log phase), coinciding with acidification of the growth medium to just below pH 6.3. Thus, it appeared that the combination of temperature and pH was responsible for activating *psaA* transcription. However, the pH of the growth medium at 26°C never got below 6.5, so we could not rule out the possibility that low pH, rather than temperature, was the activating signal. Furthermore, as the pH of the growth medium at 37°C did not drop below 6.3 until 6 h, it was also possible that growth phase played a role in the activation of *psaA* transcription. To test these possibilities, cultures of WT containing the *psaA-gfp* plasmid were grown in BHI broth buffered at pH 7.3, 6.7, or 6.3 (Fig. 1C). During growth at 37°C and pH 6.3 (37°C/pH 6.3), *psaA-gfp* activity was detected within 2 h, suggesting that low pH, rather than growth phase, induced the expression of *psaA*. The expression of *psaA* remained minimal throughout growth at 37°C/pH 7.3, but an intermediate level of expression was detected at 37°C/pH 6.7. Minimal *psaA-gfp* expression was detected after growth at 26°C/pH 6.3 (Fig. 1D). Importantly, levels of bacterial growth were not significantly different between the conditions tested (data not shown).

To determine if PsaA production corresponded with *psaA-gfp* expression, the WT was grown at 37°C in unbuffered BHI broth and in BHI broth buffered to pH 6.3 and pH 6.7, and PsaA was analyzed via Western blotting using an anti-PsaA antibody (Fig. 1E). In unbuffered BHI broth, PsaA was detectable shortly after *psaA-gfp* expression was detected (8 h) in late log phase of growth. In contrast, PsaA was detected within 2 h of growth at 37°C/pH 6.3. Thus, the production of PsaA exhibited a similar pattern of regulation in response to pH as *psaA* transcription. Based on these results, 37°C/pH 6.3 was defined as an inducing growth condition for *psaA* transcription (and PsaA production), whereas 37°C/pH 7.3 and 26°C/pH 6.3 were defined as noninducing growth conditions. Thus, high temperature and low pH provide discrete signals that are both required to activate *psaA* transcription.

**FIG 2:**
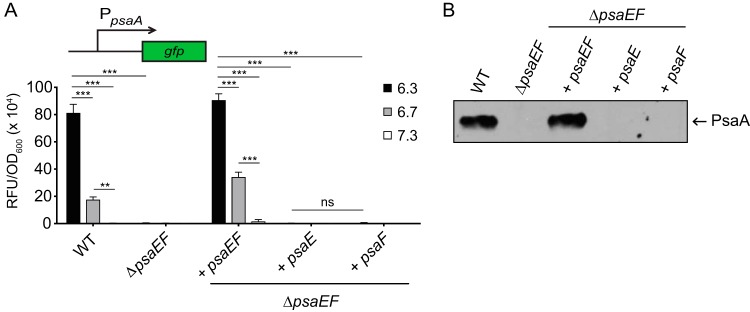
*psaE* and *psaF* are required for *psaA* transcription and production of PsaA. WT and mutant strains of Y. pestis were grown at 37°C in buffered BHI broth, and *psaA* transcription (A) and PsaA (B) were analyzed as indicated in Materials and Methods. (A) The *psaA-gfp* reporter (pEW102) was introduced into WT (YP6), the Δ*psaEF* mutant (YPA18), and derivatives of the Δ*psaEF* mutant containing *psaEF* (YPA260), *psaE* only (YPA265), or *psaF* only (YPA279), and expression was determined. Each bar represents the mean RFU/OD_600_, and error bars represent standard deviations. Each sample was assayed in biological triplicate, and at least three independent experiments were performed. Bars represent the different pHs used (see key). ***, *P* < 0.0001; **, *P* < 0.01; ns, not significant by one-way analysis of variance (ANOVA) and Tukey’s multiple-comparison test. (B) Whole-cell lysates of the strains from panel A lacking the *psaA-gfp* plasmid and grown at 37°C/pH 6.3 were prepared, and PsaA was analyzed via Western blotting, as indicated in Materials and Methods. At least three independent experiments were performed. Data presented are from a representative experiment.

To characterize the role of PsaE and PsaF in the expression of *psaA* in Y. pestis, the *psaA-gfp* plasmid was introduced into the Δ*psaEF* mutant (YPA18), and expression was compared between the Δ*psaEF* mutant and WT strains (Fig. 2A). Consistent with previous data in Y. pseudotuberculosis (27), expression of *psaA-gfp* was not detected in the Δ*psaEF* mutant even at 37°C/pH 6.3 (inducing condition), indicating that PsaE and/or PsaF is required for *psaA* transcription in Y. pestis. The *psaE* and *psaF* genes were introduced into the Δ*psaEF* mutant at the native site of the chromosome (YPA260). Complementation with both *psaE* and *psaF* restored the expression of *psaA* to WT levels (Fig. 2A), indicating that the loss of *psaA* expression in the Δ*psaEF* mutant was due to the deletion of *psaEF*. The complementation of either *psaE* (YPA265) or *psaF* (YPA279) alone was not sufficient to restore *psaA* transcription (Fig. 2A). Similarly, both *psaE* and *psaF* were required for the production of PsaA, as PsaA was undetectable when *psaE* or *psaF* alone was introduced into the Δ*psaEF* mutant (Fig. 2B). These results suggest that both *psaE* and *psaF* are required for *psaA* transcription, and thus PsaA production, in Y. pestis.

### Temperature and pH impact PsaE and PsaF levels through separate posttranscriptional mechanisms.

**FIG 3:**
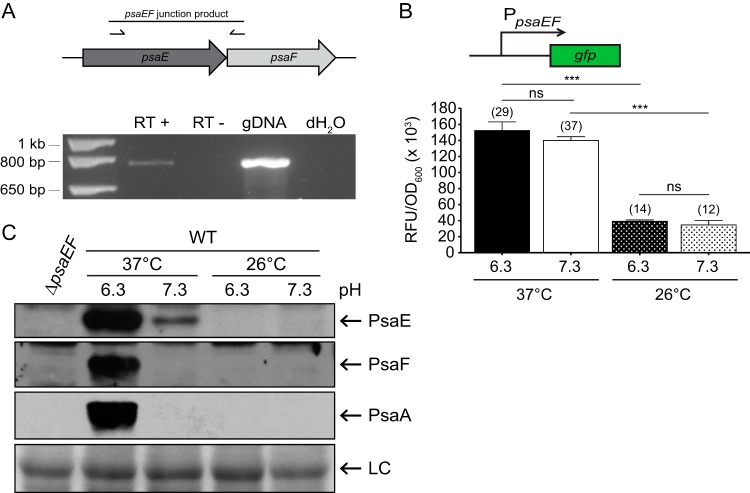
Levels of PsaE and PsaF are impacted by temperature and pH. (A) Diagram of the *psaEF* locus showing the location of primers and the predicted PCR product used to analyze *psaEF* cotranscription via RT-PCR. Templates were as follows: RT+, cDNA; RT−, no reverse transcriptase (negative control); gDNA, YP6 gDNA; dH_2_O, no template (negative control). (B) WT containing a *psaEF* transcriptional reporter plasmid (pJC126, *psaEF-gfp*) was grown at 37°C and 26°C in buffered BHI broth, and the RFU/OD_600_ was determined under each condition, as described in Materials and Methods. The number depicted over each bar indicates fold change in *psaEF-gfp* expression in the WT compared to the expression from the vector control under the given condition. Each bar represents the mean RFU/OD_600_, and error bars represent standard deviations. Each sample was assayed in biological triplicates, and at least three independent experiments were performed. ***, *P* < 0.0001; ns, not significant by one-way ANOVA and Tukey’s multiple-comparison test. (C) Whole-cell lysates of WT Y. pestis grown at 37°C and 26°C in buffered BHI broth were prepared, and PsaE, PsaF, and PsaA were analyzed via Western blotting, as indicated in Materials and Methods. As a control, whole-cell lysates of the Δ*psaEF* mutant grown at 37°C in BHI broth buffered to pH 6.3 were also analyzed. Prior to probing with antibody, a PVDF membrane was stained with Ponceau S to assess loading (LC, loading control). At least three independent experiments were performed. The data presented are from a representative experiment.

*psaE* and *psaF* coding sequences overlap by 4 bp and are predicted to be cotranscribed from a promoter upstream of *psaE*. To test this, RNA was isolated from the WT strain grown at 37°C/pH 6.3, and reverse transcription-PCR (RT-PCR) was used to analyze the *psaE-psaF* junction. Using a single set of primers internal to *psaE* and *psaF*, a product was obtained from a template that was subjected to reverse transcription, indicating that these genes are indeed cotranscribed (Fig. 3A). In Y. pseudotuberculosis, the expression of *psaE* and *psaF* is not significantly affected by temperature or pH (27). To determine if this is also true in Y. pestis, a *psaEF-gfp* transcriptional reporter plasmid (pJC126) was introduced into the WT, and expression was measured after growth at 26°C and 37°C in BHI broth buffered at pH 6.3 or 7.3. The expression of *psaEF-gfp* was detected under all four growth conditions (Fig. 3B). While expression at 37°C was moderately higher than that at 26°C (3-fold), there was no significant difference between expression levels at pH 6.3 and pH 7.3 at either temperature. Notably, *psaEF-gfp* expression was much higher than in the WT containing the vector control under the four conditions, suggesting that the expression of *psaEF* occurred under all four growth conditions and, unlike the expression of *psaA*, was not largely impacted by pH. Thus, the temperature- and pH-dependent regulation of *psaA* expression is not dictated by transcriptional regulation of *psaEF* in Y. pestis.

Based on experiments in Y. pseudotuberculosis, Yang and Isberg speculated that PsaE and PsaF are subject to posttranscriptional regulation in response to temperature and pH (27). While transcription of *psaE* and *psaF* in Y. pseudotuberculosis occurred under all growth conditions tested, PsaE and/or PsaF protein levels were not determined (27). Therefore, to investigate if the levels of the PsaE and PsaF proteins were impacted by temperature and/or pH, antibodies were generated against PsaE and PsaF, and whole-cell lysates of the WT grown under inducing and noninducing conditions were analyzed by Western blotting (Fig. 3C). PsaE and PsaF were detected only in samples grown at 37°C/pH 6.3 and corresponded with the detection of PsaA. Although the expression of *psaEF-gfp* at 37°C/pH 6.3 was similar to that at pH 7.3, PsaE levels were consistently lower at 37°C/pH 7.3, and PsaF was never detected at 37°C/pH 7.3. Furthermore, neither PsaE nor PsaF was detected at 26°C at either pH. Thus, the production of PsaE and PsaF appears to be regulated by posttranscriptional mechanisms, and the mechanisms appear to impact PsaE and PsaF differently in response to temperature and pH. Since the detection of PsaA requires PsaE and PsaF, these mechanisms indirectly impact downstream PsaA production.

### Temperature-dependent translation of *psaE* is mediated by the *psaE* 5′ UTR.

**FIG 4:**
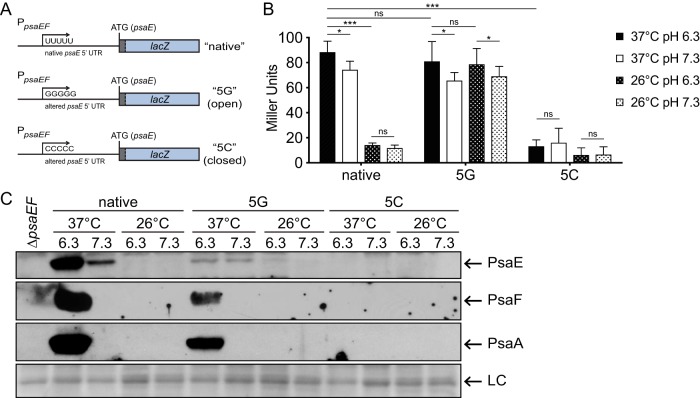
*psaE* translation requires high temperature and is influenced by uridine residues in the *psaE* mRNA 5′ UTR. (A and B) Translational reporters expressing *lacZ* under the control of the native *psaEF* promoter fused to either the native *psaE* 5′ UTR (*psaE*^native^-*lacZ*) or *psaE* 5′ UTR mutant variants with the 5G (*psaE*^5G^-*lacZ*) or 5C (*psaE*^5C^-*lacZ*) nucleotide alterations (A) were constructed to examine regulation in WT Y. pestis (B). (C) The same *psaE* 5′ UTR variants fused to the *psaEF* promoter were used to express *psaEF* in Y. pestis. (A) Diagram of translational reporters containing the native *psaEF* promoter fused to the native *psaE* 5′ UTR or altered *psaE* 5′ UTR variants with nucleotide substitutions. (B) Y. pestis mutants containing *lacZ* reporters (depicted in panel A) at the Tn*7* site were grown under the indicated condition, and β-galactosidase activity was analyzed for each strain, as indicated in Materials and Methods. Each bar represents the mean value of Miller units, and error bars represent standard deviations. Each sample was assayed in biological triplicate, and the data presented are from a representative experiment. ***, *P* < 0.0001; *, *P* < 0.05; ns, not significant by two-way ANOVA and Tukey’s multiple-comparison test. (C) Whole-cell lysates of Y. pestis strains expressing *psaEF* from the *psaE*^native^ 5′ UTR (native), the *psaE*^5G^ 5′ UTR (5G), or the *psaE*^5C^ 5′ UTR (5C) were prepared, and PsaE, PsaF, and PsaA were analyzed via Western blotting, as indicated in Materials and Methods. Prior to probing with antibody, a PVDF membrane was stained with Ponceau S to assess loading in each lane (LC; loading control). At least three independent experiments were performed. Data presented are from a representative experiment.

The absence of PsaE and PsaF at 26°C, despite the high level of transcription, suggested that temperature may regulate the translation of *psaE* and *psaF*. To investigate this, we generated a *psaE*^native^-*lacZ* translational reporter (pJQ021) containing *lacZ* fused to the native *psaEF* promoter, 5′ untranslated region (UTR), and *psaE* start codon, including the first 18 nucleotides of the *psaE* coding sequence, such that the translation of *lacZ* was directly dependent on the *psaE* 5′ UTR (Fig. 4A, “native”). This reporter was introduced into the Δ*lacZ* mutant (YPA87) at the Tn*7* site, and this reporter strain (YPA355) was grown under the four conditions tested above. β-Galactosidase activity from *psaE*^native^-*lacZ* was significantly higher at 37°C than at 26°C (6-fold), suggesting that translation initiation occurred more readily at 37°C (Fig. 4B). At both temperatures, β-galactosidase activity was only moderately lower at pH 7.3 than at pH 6.3, so translation initiation did not appear to be affected by pH. These results suggest that the *psaE* 5′ UTR mediates temperature-dependent translation.

**FIG 5:**
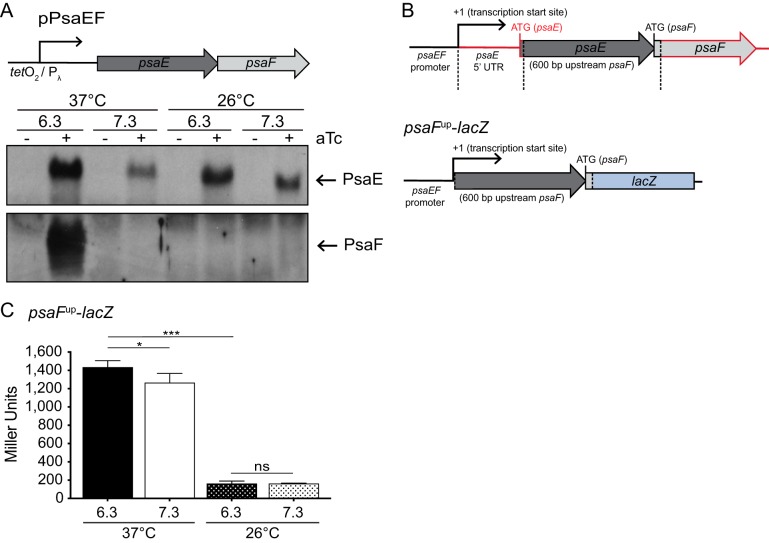
*psaF* translation requires high temperature and is regulated independently of the *psaE* 5′ UTR. The *psaEF* coding sequence was cloned into a *tet*-inducible expression vector (pPsaEF). (A) The Δ*psaEF* mutant carrying pPsaEF was grown at 37°C and 26°C in buffered BHI broth in the presence or absence of 50 ng/ml ATc, and whole-cell lysates were prepared and used to analyze PsaE and PsaF via Western blotting, as indicated in Materials and Methods. P_λ_, phage lambda promoter. (B) Diagram of sequence used to construct the *psaF* translational reporter (pJQ043, *psaF*^up^-*lacZ*); the *psaEF* promoter was ligated directly to sequences upstream of *psaF*. The fragments outlined in red were excluded from the reporter. (C) The *psaF*^up^-*lacZ* reporter was introduced at the Tn*7* site in the Y. pestis Δ*lacZ* mutant (YPA87), and this strain was grown at 37°C or 26°C in buffered BHI broth. β-Galactosidase activity was analyzed as indicated in Materials and Methods. Bar graphs represent mean values of Miller units, and error bars represent standard deviations. ***, *P* < 0.0001; *, *P* < 0.05; ns, not significant by one-way ANOVA and Tukey’s multiple-comparison test. At least three independent experiments were performed. Data presented are from a representative experiment.

Recent analysis of the RNA “structurome” in Y. pseudotuberculosis suggests the existence of numerous RNA thermometers mediating temperature-dependent translation (32). The transcription start site of *psaEF* mRNA has been mapped (33), and within the *psaEF* 5′ UTR, there is a string of uridine residues (+7 to +11) that resemble a fourU RNA thermometer (34). FourU thermometers regulate the translation of downstream genes by modulating a temperature-responsive mRNA structure formed by imperfect base pairing of uridine residues to residues in the ribosome binding site (RBS) (35), and nucleotide substitutions that disrupt this base pairing can impact the translation of downstream genes (36, 37). Thus, we hypothesized that the string of uridine residues within the *psaE* 5′ UTR functioned as an RNA thermometer and that mutating these residues would disrupt the temperature-dependent translation mediated by the *psaE* 5′ UTR. To test this, we generated two additional translational reporters in which the string of uridine residues in the *psaE* 5′ UTR was altered. In one construct, the nucleotides were all changed to guanine (5G), which would potentially prevent base pairing with guanine residues in the RBS (pJQ028) (Fig. 4A and 5G). In the second construct, the nucleotides were all changed to cytosine (5C), which would potentially strengthen base pairing with guanine residues in the RBS (pJQ027) (Fig. 4A and 5C). These reporters were integrated into the Δ*lacZ* mutant at the Tn*7* site to generate *psaE*^5G^-*lacZ* (YPA359) and *psaE*^5C^-*lacZ* (YPA357) reporter strains, respectively. These strains were grown under the four growth conditions as described before, and β-galactosidase activity was measured (Fig. 4B). The activity of *psaE*^5G^-*lacZ* was similar at both 37°C and 26°C, and expression of *psaE*^5G^-*lacZ* was significantly higher than *psaE*^native^-*lacZ* at 26°C. There was only a modest reduction in the expression of *psaE*^5G^-*lacZ* at pH 7.3 compared to pH 6.3, suggesting that translation of *psaE*^5G^-*lacZ* occurred under all four growth conditions. Conversely, *psaE*^5C^-*lacZ* had low β-galactosidase activity even at 37°C. These data suggest that the 5′ UTR of *psaE* mediates temperature-dependent translation and that uridine residues within the 5′ UTR contribute to this regulatory mechanism.

As the *lacZ* translational reporters demonstrated that temperature-dependent translation in the *psaE* 5′ UTR sequence is influenced by the uridine residue motif, we wanted to determine if PsaE and PsaF are produced at 26°C when *psaE* and *psaF* were encoded downstream of the *psaE*^5G^ 5′ UTR. To test this, the *psaE* and *psaF* genes were fused to the *psaE*^5G^ 5′ UTR and native promoter and introduced into the Δ*psaEF* mutant at the native site on the chromosome, effectively replacing the native *psaE* 5′ UTR, to generate the *psaE*^5G^ strain (YPA361). PsaE, PsaF, and PsaA were analyzed by Western blotting following growth of the WT, *psaE*^5G^ mutant, and *psaE*^5C^ mutant under the four growth conditions (Fig. 4C). At 26°C/pH 6.3, where PsaE is not normally detected in WT, low levels of PsaE were detected in the *psaE*^5G^ mutant, indicating that PsaE can be produced at 26°C. However, PsaF and PsaA could still be detected only in cultures of the *psaE*^5G^ mutant grown at 37°C/pH 6.3, indicating that synthesis of PsaE at 26°C was not sufficient for production of PsaF or PsaA at 26°C. Notably, PsaE levels were relatively low under all conditions in the *psaE*^5G^ mutant, indicating that the stability of PsaE may be altered, even at 37°C/pH 6.3. Curiously, the levels of PsaF (and PsaA) appeared to be moderately low in the *psaE*^5G^ mutant (relative to *psaE*^native^) at 37°C/pH 6.3, corresponding with the slightly lower level of PsaE. No PsaE, PsaF, or PsaA was detected under any of the same four growth conditions in the *psaE*^5C^ mutant, indicating that PsaE, PsaF, and PsaA were not produced under any growth conditions in this mutant. Taken together, these data suggest that the 5′ UTR of *psaE* regulates the translation of *psaE* in response to temperature, but PsaF is influenced by temperature and pH through additional mechanisms.

### Translation of *psaF* is temperature dependent.

To determine if the production of PsaF was affected by temperature or pH independently of the *psaE* 5′ UTR, the *psaEF* coding sequences were cloned into an inducible expression construct such that the expression of *psaEF* could be induced by the addition of anhydrotetracycline (ATc). Importantly, the *psaE* 5′ UTR was not present upstream of *psaE* in this construct and thus would not influence PsaE or PsaF production. This plasmid (pPsaEF) was introduced into the Δ*psaEF* mutant; this strain was grown under all four growth conditions in the presence and absence of ATc, and cell lysates were analyzed by Western blotting (Fig. 5A). PsaE was detected under all four growth conditions when ATc was added, even at low temperature. Yet, despite the presence of PsaE under all four conditions, PsaF was detectable only in samples grown at 37°C/pH 6.3, as seen in the *psaE*^5G^ mutant. These data indicate that the regulation of PsaF production by temperature and pH occurs through a mechanism distinct from the *psaE* 5′ UTR.

While it was possible that the translation of *psaF* was regulated by temperature and/or pH, it was also possible that the stability of PsaF was impacted by these signals. To determine whether translation initiation of *psaF* was affected by temperature and/or pH, we made a *psaF*^up^-*lacZ* translational reporter (pJQ043) containing *lacZ* fused to sequence upstream of *psaF* (*psaE* coding sequence), the *psaF* start codon including the first 30 nucleotides of the *psaF* coding sequence, and the native *psaEF* promoter (up to the *psaEF* transcription start site) (Fig. 5B). Importantly, this expression construct lacks the native *psaE* 5′ UTR (including the *psaE* RBS) and *psaE* start codon; therefore, the translation of *lacZ* is directly dependent upon sequences upstream of the *psaF* start codon. Since the *psaEF* promoter was active under all growth conditions tested, we reasoned that any significant differences in β-galactosidase activity would be dependent on the translation of *psaF*. This reporter was introduced into the Δ*lacZ* mutant at the Tn*7* site to generate the *psaF*^up^-*lacZ* strain (YPA424). This strain was grown under the four growth conditions as described above, and β-galactosidase activity was measured (Fig. 5C). β-Galactosidase activity was higher (8-fold) at 37°C than at 26°C, and while there was a slight difference in expression between pH 6.3 and pH 7.3, translation did not appear to be largely impacted by pH. Thus, the translation of *psaF* requires high temperature and this regulation is mediated by the sequence upstream of *psaF*. However, control of PsaF production in response to temperature did not account for observed differences in PsaF levels in response to pH.

### PsaF influences *psaA* transcription by affecting PsaE levels.

**FIG 6:**
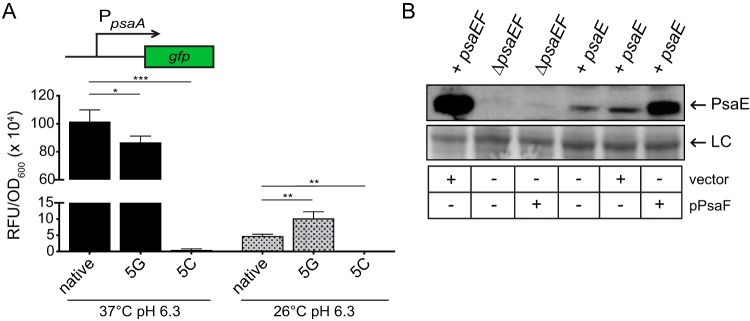
Expression of *psaA* and enhanced PsaE stability correspond with PsaF. (A) The *psaA-gfp* reporter was introduced into derivatives of the Δ*psaEF* mutant expressing *psaEF* from the *psaE*^native^ 5′ UTR (YPA260, native), the *psaE*^5G^ 5′ UTR (YPA361, 5G), and the *psaE*^5C^ 5′ UTR (YPA360, 5C); these strains were grown at 37°C and 26°C in pH 6.3 buffered BHI broth, and reporter expression was determined as described in Materials and Methods. Each bar represents the mean RFU/OD_600_, and error bars represent standard deviations. Each sample was assayed in biological triplicate. ***, *P* < 0.0001; **, *P* < 0.01; *, *P* < 0.05 by one-way ANOVA and Dunnett’s multiple-comparison test. (B) The *psaF* complementation plasmid (pPsaF) or vector was introduced into the Δ*psaEF* mutant (YPA18) or derivatives of the Δ*psaEF* mutant expressing *psaEF* (YPA260, *psaEF*^+^) or *psaE* only (YPA265, *psaE*^+^). These strains were grown at 37°C in pH 6.3 buffered BHI broth, and whole-cell lysates were prepared and used to analyze PsaE via Western blotting, as indicated in Materials and Methods. Prior to probing with antibody, a PVDF membrane was stained with Ponceau S to assess loading in each lane (LC, loading control). At least three independent experiments were performed. Data presented are from a representative experiment.

While PsaE is predicted to be the direct transcriptional activator of *psaA* transcription, the role of PsaF is less clear. While *psaF* is required for *psaA* transcription (Fig. 2A), in the *psaE*^5G^ mutant (where *psaF* is present but PsaF is not produced at 26°C/pH 6.3), PsaA was not detected in samples grown at 26°C/pH 6.3 despite low levels of PsaE. These data suggest that the production of PsaF, in addition to PsaE, is required for *psaA* expression. To further dissect this phenomenon and investigate the role of PsaF in the regulation of *psaA* transcription, the *psaA-gfp* plasmid was introduced in the *psaE*^native^ (YPA260), *psaE*^5G^ (YPA361), and *psaE*^5C^ (YPA360) strains, and expression was analyzed after growth at 37°C and 26°C in BHI broth buffered to pH 6.3 (Fig. 6A). High levels of *psaA-gfp* expression occurred in the *psaE*^native^ and *psaE*^5G^ strains at 37°C/pH 6.3, while no expression occurred in the *psaE*^5C^ mutant. These data correspond with the absence of PsaE, PsaF, and PsaA in the *psaE*^5C^ mutant and the presence of these proteins in the *psaE*^native^ and *psaE*^5G^ strains under this growth condition. The expression of *psaA-gfp* was slightly lower in the *psaE*^5G^ strain than in the *psaE*^native^ strain, corresponding with the slightly lower levels of PsaA previously noted in the *psaE*^5G^ strain (Fig. 4C). Conversely, minimal *psaA-gfp* expression occurred in the *psaE*^native^ and *psaE*^5G^ strains at 26°C/pH 6.3, while expression was not detected in the *psaE*^5C^ strain. Despite *psaA-gfp* expression being low, expression in the *psaE*^5G^ strain was 2-fold higher than in the *psaE*^native^ strain. This moderate increase in *psaA-gfp* expression corresponded with the presence of low levels of PsaE in the *psaE*^5G^ strain and the absence of PsaE in the *psaE*^native^ strain under these growth conditions (Fig. 4C). These data further support the hypothesis that PsaE acts as a direct transcriptional activator of *psaA* transcription; however, the presence of PsaE alone is not sufficient for high-level expression of *psaA*, indicating that PsaF is required for maximal activation of *psaA* transcription.

The topologies of PsaE and PsaF resemble those of the ToxR-ToxS and TcpP-TcpH regulatory protein pairs in Vibrio cholerae (27, 29, 30, 38). ToxS and TcpH enhance the stability of ToxR and TcpP, respectively (39, 40), and since PsaE levels are reduced in the absence of PsaF, we wondered if PsaF influenced PsaE stability. To test this, we constructed a plasmid expressing *psaF* under the control of the *psaEF* promoter and the *psaF* 5′ UTR (pPsaF; same sequence driving the *psaF*^up^-*lacZ* reporter in Fig. 5B). This plasmid and a vector control plasmid were introduced into the strain expressing only *psaE* at the native site (YPA265), and PsaE levels were analyzed in this strain grown at 37°C/pH 6.3 (Fig. 6B). As previously noted, when both *psaE* and *psaF* are present, PsaE was detected at high levels. However, in the absence of *psaF*, PsaE was detected only at low levels, suggesting that PsaF plays a role in stabilizing PsaE. When pPsaF was introduced into YPA265, generating a strain that contains *psaE* on the chromosome and *psaF* on a plasmid, higher levels of PsaE were detected than in the vector control strain lacking pPsaF. Curiously, lower levels of PsaE were detected when *psaF* was expressed in *trans* than with native chromosomal expression, thus suggesting that cotranscription of *psaE* and *psaF* may be required for maximum levels of PsaE. Despite the slight reduction from *trans* complementation of *psaF* (compared to WT), these data indicate that PsaF increases levels of PsaE, and this likely occurs through enhanced stability.

## DISCUSSION

**FIG 7:**
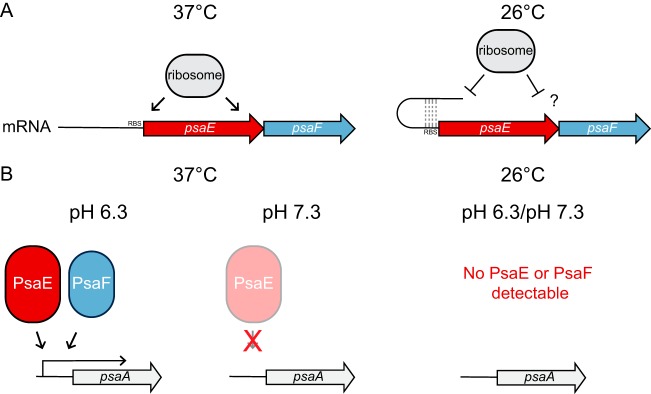
Working model of the regulation of *psaE*, *psaF*, and *psaA*. (A) Cotranscription of *psaE* and *psaF* occurs at 37°C and 26°C, while the translation of both *psaE* and *psaF* is temperature dependent. The translation of *psaE* and *psaF* is mediated by separate mechanisms. (B) Levels of PsaE and PsaF are pH dependent at 37°C. High levels of PsaE and PsaF are detected at 37°C/pH 6.3. Low levels of PsaE and no PsaF are detected at 37°C/pH 7.3. Both PsaE and PsaF are required for *psaA* transcription. The “X” indicates that PsaE is unable to activate *psaA* transcription at pH 7.3.

As a vector-borne pathogen, Y. pestis moves between a flea vector and mammalian host and must use environmental signals as cues to regulate the expression of key virulence determinants that aid in immune evasion and survival within the host tissue. The Y. pestis pH 6 antigen (PsaA) is a virulence factor with an unusual expression pattern that requires high temperature and low pH (15, 17–19, 23). It is well established that the combination of high temperature and acidic pH is required for *psaA* transcription and PsaA production in Y. pestis, but the underlying mechanisms have remained elusive (23, 24). Here, we show that both *psaA* transcription and PsaA production occur rapidly when Y. pestis is grown at high temperature in medium buffered to low pH (37°C/pH 6.3). By utilizing buffered medium for growth, we ruled out the possibility that expression of *psaA* is impacted by the growth phase of the bacteria rather than by low pH. We also investigated the roles of PsaE and PsaF, key transcriptional regulators of *psaA*, to understand how temperature and pH influence *psaA* expression. We propose a model in which temperature and pH impact PsaE and PsaF levels and, thus, the expression of *psaA* (Fig. 7).

Consistent with findings in Y. pseudotuberculosis (27), we show that both *psaE* and *psaF* are required for *psaA* transcription in Y. pestis and that the transcription of *psaE* and *psaF*, unlike *psaA*, is not affected by pH and only moderately impacted by temperature. These results support previous speculation that the function and/or levels of PsaE and PsaF are subject to posttranscriptional regulation in Y. pseudotuberculosis (27) and led us to investigate how temperature and pH influence PsaE and PsaF in Y. pestis. Prior to this study, the direct detection of PsaE and PsaF had not been reported. By generating antibodies that recognize endogenous PsaE and PsaF protein, we showed that the levels of both PsaE and PsaF are influenced by temperature and pH. Neither PsaE nor PsaF was detected after growth at low temperature (26°C), corresponding with the absence of *psaA* transcription and, thus, PsaA. Intriguingly, PsaE and PsaF appear to have different sensitivities to pH; PsaE is detectable at low levels at 37°C/pH 7.3, whereas we are unable to detect PsaF at 37°C/pH 7.3. Reduced levels of PsaE (and absence of PsaF) at 37°C/pH 7.3 also correspond with the absence of both the PsaA protein and *psaA* promoter activity. Since the presence of PsaA at 37°C/pH 6.3 corresponds with the presence of both PsaE and PsaF, understanding how temperature and pH impact PsaE and PsaF levels became the focus of this study.

Our data suggest that the translation of both *psaE* and *psaF* is temperature dependent and regulated by sequences upstream of each gene. Our finding that the 5′ UTR of *psaE* regulates translation initiation of *psaE* in response to a temperature upshift resembles the recently described mechanism regulating temperature-dependent synthesis of LcrF, a major virulence regulator of the type III secretion system (T3SS) in *Yersinia* spp. (37, 41). Similar to our findings with PsaE/PsaF, the synthesis of LcrF in Y. pestis is significantly increased at high temperature and corresponds with the expression of type III secretion system (T3SS) genes regulated by LcrF (42, 43). In Y. pseudotuberculosis, *lcrF* translation was shown to be regulated by a fourU RNA thermometer located in the mRNA upstream of *lcrF* (37). Initially characterized for the regulation of bacterial heat shock genes in response to temperature (44), recently, RNA thermometers have been identified as a mechanism to regulate the expression of virulence genes in pathogenic bacteria (36, 37, 45, 46). Additionally, a recent study of the structurome in Y. pseudotuberculosis suggests that these elements may be extensively utilized by *Yersinia* spp. (32). By mutating the uridine residues within the *psaE* 5′ UTR, we were able to overcome the thermal regulation of *psaE* translation to inhibit the detection of PsaE at 37°C (*psaE*^5C^) and detect low levels of PsaE at 26°C (*psaE*^5G^). Yet, despite the presence of PsaE at 26°C in the *psaE*^5G^ mutant, the detection of PsaF and PsaA remains limited to 37°C/pH 6.3. When *psaEF* was expressed using an inducible expression construct (pPsaEF; YPA366), in which *psaE* is expressed without the native *psaE* 5′ UTR, PsaE was present at high levels at 37°C and 26°C, but PsaF was present only at 37°C/pH 6.3. Thus, the native *psaE* 5′ UTR imparts thermal regulation on *psaE* translation, which appears to explain why PsaE is absent at 26°C in the WT.

Differences in the activity of the *psaF*^up^-*lacZ* translational reporter at 37°C and 26°C indicated that the sequence upstream of *psaF* (*psaE* coding sequence) imparts temperature-dependent regulation on *psaF* translation. This additional layer of thermal regulation likely explains why PsaF remains absent in the *psaE*^5G^ mutant at 26°C, despite the ability to induce the synthesis of PsaE. The discrete regulation of both PsaE and PsaF synthesis by high temperature reveals that Y. pestis incorporates multiple mechanisms to ensure that the production of PsaA is specific for high temperature. Similarly, when thermal regulation of *lcrF* translation was overcome by disrupting the RNA thermometer upstream of *lcrF* in Y. pseudotuberculosis, downstream Yop production remained specific for high temperature, despite detectable LcrF at low temperature (37), thus suggesting the presence of multiple layers of regulation. While it is possible that the uridine residues (+7 to +11) in the *psaE* mRNA 5′ UTR function as a fourU RNA thermometer, additional studies will be needed to determine if the *psaE* 5′ UTR influences folding of *psaEF* mRNA in response to temperature in a manner similar to characterized fourU RNA thermometers (34).

The topologies of PsaE and PsaF are similar to that of the ToxR/ToxS-like family of transcriptional regulatory protein pairs in V. cholerae (29, 38). Members of this protein family serve as key regulators of virulence gene expression (28–31, 39, 40, 47–51), and our data indicate that PsaE and PsaF are key regulators of *psaA* transcription in Y. pestis. ToxR-like proteins are predicted to sense environmental signals and influence downstream gene expression, as both sensing (periplasmic) and DNA-binding (cytoplasmic) domains are contained within a single protein. However, the mechanisms by which ToxR-like proteins integrate signal sensing and downstream gene activation are not well understood. The membrane spanning topology of ToxR-like proteins offers an unusual potential to function as a one-component signal transduction system, as seen with the acid-sensing regulator CadC (52). Intriguingly, the stability and/or function of other ToxR-like family proteins, such as TcpP, and ToxR itself, are affected by a second effector protein (TcpH and ToxS, respectively) (31, 39, 40, 49, 53, 54). Characteristic of ToxR-like proteins, the N-terminal cytoplasmic domain of PsaE contains a winged helix-turn-helix DNA-binding motif resembling the OmpR family of response regulators (16). While an interaction of PsaE with the *psaA* promoter has not been demonstrated, it is predicted that PsaE directly activates *psaA* transcription. However, as PsaA is not detected unless both PsaE and PsaF are present, PsaE alone is not sufficient to activate *psaA* transcription. Thus, like ToxR and TcpP, PsaE requires a partner protein to regulate gene expression.

The N terminus of PsaF is thought to be anchored to the inner membrane (27), and therefore, PsaF is likely not directly involved in binding to *psaA* promoter DNA. We found that PsaE levels are influenced by PsaF; thus, it is plausible that the primary role of PsaF is to enhance PsaE stability to allow for *psaA* transcription. In support of this, high levels of PsaE and PsaA correspond with the presence of PsaF. However, there is evidence to suggest that PsaF also influences PsaE function. When the WT is grown at 37°C/pH 7.3, low levels of PsaE are detected, but PsaF is not detected. Under these conditions, the level of *psaA* expression is just as low as in the Δ*psaEF* mutant, further indicating that PsaE alone cannot activate *psaA* transcription. These data clearly indicate that both PsaE and PsaF are required for the expression of *psaA* and suggest that the role of PsaF may be to promote the stability and/or function of PsaE.

The role of pH as an environmental signal for the expression of *psaA* remains unclear, as it is not known when Y. pestis encounters an acidic pH during mammalian infection. While it has been speculated that a macrophage phagosome may provide the low-pH environment that is necessary for *psaA* transcription (16), the expression of *psaA* in host cells during a mammalian infection has not been demonstrated. Strikingly, little is known about mechanisms that bacteria utilize to sense pH and influence gene expression. Our work suggests that Y. pestis may utilize PsaE and PsaF to sense changes in pH to regulate the expression of *psaA*. Despite thermal regulation of *psaE* and *psaF* translation, the translation of *psaE* and *psaF* is not influenced by changes in pH. However, the levels of both PsaE and PsaF do show a significant change in response to pH, suggesting that there are additional posttranslational pH-dependent mechanisms regulating PsaE and PsaF. Since PsaE levels are affected by PsaF, it is tempting to speculate that PsaF contributes to pH-dependent stability of both proteins. To address this, we are currently investigating the mechanisms by which pH regulates PsaE and PsaF. Together, our data suggest that Y. pestis utilizes temperature and pH to influence levels of PsaE and PsaF, two key transcriptional regulatory proteins of *psaA* in Y. pestis. The temperature-dependent regulation of *psaE* and *psaF* translation, in addition to the regulation of PsaE and PsaF by pH, allows Y. pestis to precisely control the expression of *psaA* through multiple environmental signals.

## MATERIALS AND METHODS

### Bacterial strains and growth conditions.

All bacterial strains and plasmids used in this study are listed in Table 1. Y. pestis CO92/pCD1^−^ (YP6) was cultivated on brain heart infusion (BHI) agar (BD Biosciences, Bedford, MA) at 26°C for 48 h and in BHI broth cultures grown with aeration at 26°C or 37°C. E. coli strains were cultivated on Luria-Bertani (LB) agar (BD Biosciences) at 37°C overnight and in liquid cultures with aeration at 37°C or 26°C. When indicated, bacteria were grown in BHI broth that was adjusted and buffered to the appropriate pH. BHI broth was buffered with 100 mM MES [2-(*N*-morpholino)ethanesulfonic acid; Sigma] and then adjusted to pH 6.3 or 6.7, or it was buffered with 100 mM MOPS [3-(*N*-morpholino)propanesulfonic acid; Fisher Scientific] and then adjusted to pH 7.3 and filter sterilized. When necessary, antibiotics were added to the growth medium at the following concentrations: kanamycin (Kan), 50 μg/ml; carbenicillin (Carb), 100 μg/ml; and irgasan (Irg), 2 μg/ml. For the expression of genes cloned into pMWO-005, 50 ng/ml anhydrous tetracycline (ATc) was added to the liquid medium when strains were subcultured.

**TABLE 1:** Bacterial strains and plasmids used in this study

Strain or plasmid	Description^*a*^	Reference
Strains		
*E. coli*		
DH5α	F^−^ ϕ80Δ*lacZ*M15 Δ(*lacZYA-argF*)*U169 deoP recA1 endA1 hsdR17*(r_K_^−^ m_K_^−^)	Invitrogen
S17-1 λ*pir*	*recA thi pro hsdR hsdM*^+^ RP4::2-Tc::Mu::Km Tn*7* λ*pir* lysogen Tp^r^ Str^r^	63
*Y. pestis*		
YP6	CO92/pCD1^−^	17
YPA18	YP6 Δ*psaEF*	This work
YPA87	YP6 Δ*lacZ*	This work
YPA260	YPA18 with pEW104 at the native site	This work
YPA265	YPA18 with pEW105 at the native site	This work
YPA279	YPA18 with pEW106 at the native site	This work
YPA355	YPA87 with *psaE*^native^-*lacZ* from pJQ021 at the Tn*7* site	This work
YPA357	YPA87 with *psaE*^5C^-*lacZ* from pJQ027 at the Tn*7* site	This work
YPA359	YPA87 with *psaE*^5G^-*lacZ* from pJQ028 at the Tn*7* site	This work
YPA360	YPA18 with pJQ029 at the native site	This work
YPA361	YPA18 with pJQ030 at the native site	This work
YPA424	YPA87 with *psaF*^up^-*lacZ* from pJQ043 at the Tn*7* site	This work
Plasmids		
pSR47S	MobRP4 *oriR6K sacB* suicide vector, Kan^r^	55
pPROBE-AT	*gfp* reporter vector, Ap^r^	56
pPROBE-gfp[tagless]	*gfp* reporter vector, Kan^r^	56
pEW102	*psaA* promoter in pPROBE-AT	This work
pJC126	*psaEF* promoter in pPROBE-gfp[tagless]	This work
pJC306	*psaEF* flanking sequences in pSR47S	This work
pEW104	*psaEF* and flanking sequences in pSR47S	This work
pEW105	*psaEF* promoter and *psaE* in pSR47S	This work
pEW106	*psaEF* promoter and *psaF* in pSR47S	This work
pMWO-005	Low-copy-number expression vector, Kan^r^	60
pPsaEF	*psaEF* coding sequence in pMWO-005	This work
pWKS30	Cloning vector, Ap^r^	61
pPsaF	*psaEF* promoter and *psaF* coding sequence in pWKS30	This work
pUC18R6K-mini-Tn7T	R6K replicon and mini- Tn*7* delivery vector, Ap^r^	58
pEW103	Kan^r^ and FRT flanks from pKD13 cloned into pUC18R6K-mini-Tn7T	This work
pJQ003	pEW103 containing *lacZ* sequence from pFU61	This work
pJQ021	*psaEF* promoter and native *psaE* 5′ UTR in pJQ003	This work
pJQ027	*psaEF* promoter and 5C *psaE* 5′ UTR in pJQ003	This work
pJQ028	*psaEF* promoter and 5G *psaE* 5′ UTR in pJQ003	This work
pJQ029	*psaEF* and flanking regions with 5C *psaE* 5′ UTR in pSR47S	This work
pJQ030	*psaEF* and flanking regions with 5G *psaE* 5′ UTR in pSR47S	This work
pJQ043	*psaEF* promoter fused to *psaF* upstream sequence into pJQ003	This work

a Tp^r^, trimethoprim resistance; Str^r^, streptomycin resistance; Kan^r^, kanamycin resistance; Ap^r^, apramycin resistance; FRT, FLP recombination target.

### Plasmid and strain construction.

All primers used in this study are listed in Table 2. The Δ*psaEF* mutant and all mutants in which *psaE* and/or *psaF* were introduced at the native site in the Δ*psaEF* mutant were constructed via allelic exchange using the pSR47S suicide vector (55). All plasmids were constructed via Gibson Assembly (NEB), unless otherwise described, and were confirmed by sequencing.

**TABLE 2:** Primers used in this study

Primer	Sequence (5′ to 3′)^*a*^	Use^*b*^
EW1021	TTGCATGCCTGCAGGTCGACCCCTCTTCATTCATATCAGTCATC	F pEW102 5′
EW1022	AGCTCGGTACCCGGGGATCCCATTAGTGTGGTAACCGCCAGCG	R pEW102 3′
*psaEFgfp*-5′	ACGC**GTCGAC**CTGCGCTGGTACTGGGGCTGTGC	F pJC126 5′
*psaEFgfp*-3′	CGC**GGATCC**GGATAAAGCATATCTACTGTCACC	R pJC126 3′
*psaE*-del_up	ACGC**GTCGAC**GGGCTATCCATCCAGTGCTGTTATATTTGG	F pJC306 up 5′
*psaE*-del_dwn	CGC**GGATCC**GTGACTCATTTGCCCTCACCTCCCCTGATC	R pJC306 up 3′
*psaF*-del_up	CGC**GGATCC**GGGGTACAAGGAGAACATATCCATACGTCCTAT	F pJC306 down 5′
*psaF*-del_dwn	ATAAGAAT**GCGGCCGC**ATAACTCAGTCGCAGACCTATAGATAGAGAA	R pJC306 down 3′
*psaEF*compF	ATCGATCCTCTAGA**GTCGAC**ATTAACGGGGGCGCTGTCTATGG	F pEW104 5′
*psaEF*compR	GCTCTAGAACTAGT**GGATCC**ATAACTCAGTCGCAGACCTATAG	R pEW104 3′
*psaE*comp-upR	ATCTAAAATAGATTAATTTCATTGCTGTTTGCATTCCG	R pEW105 3′
*psaE*comp-dwnF	CAGCAATGAAATTAATCTATTTTAGATGACATTTTTA	F pEW105 5′
*psaF*comp-upR	TTGCTTTCATTTGCCCTCACCTCCCCTGATCTGGA	R pEW106 3′
*psaF*comp-dwnF	GGGAGGTGAGGGCAAATGAAAGCAAAATCACTTACTC	F pEW106 5′
JQ129	TACACAAAAGCTAAACAATATTTAAACAAAAGTCAACCCAG	R pJQ043 3′ internal
JQ130	ATATTGTTTAGCTTTTGTGTATCACTGTGTTGTTTTAAATAA	F pJQ043 5′ internal
JQ133	CTAGCTGCGCGGCCGCCTCGAGATATGAGAGTAAGTGATTTTGC	R pJQ043 3′
pKD13kanF	**GATATC**GTGTAGGCTGGAGCTGCTTC	F pEW103 5′
pKD13kanR	**GATATC**ATTCCGGGGATCCGTCGACC	R pEW103 3′
JQ018	GCG**CTCGAG**GCGGCCGCGCAGCTAGCGTCGACG	F pJQ003 5′
JQ019	GAGGCCT**GGTACC**GCCTCTAGAGCGGC	R pJQ003 3′
JQ047	CGATATCATGCATGAGCTCAATTAACGGGGGCGCTGTCTATGG	F pJQ021 5′
JQ048	GCTAGCTGCGCGGCCGCCTCGAACAACACAGTGACTCATTTGC	R pJQ021 3′
JQ058	CTTTTGTGTATAAAACCCCCCCAATTAAGACTCACTTATG	F pJQ027 internal 5′
JQ059	GGGGGTTTTATACACAAAAGCTAAACAATATTTAAACAAAAGTC	R pJQ027 internal 3′
JQ071	CCCCCTTTTATACACAAAAGCTAAACAATATTTAAACAAAAGTC	R pJQ028 internal 3′
JQ072	GCTTTTGTGTATAAAAGGGGGCCAATTAAGACTCACTTATG	F pJQ028 internal 5′
JQ148	TCGCCGTGAAGGTAAAGTTC	F *gyrB* internal 5′
JQ149	ATTGGTAAAGGTCTGGAAACTTGGCC	R *gyrB* internal 3′
JQ054	AGGTGCTGCTGTTAGAGTGTC	F *psaE* internal 5′
JQ101	CGGATATGTTTAATAGCAACCG	R *psaE* internal 3′
JQ103	GAAGTGAAATATGGCGATATCC	F *psaF* internal 5′
JQ104	CGTTGGCATTTTCAAACCAATACC	R *psaF* internal 3′
CPL100	CGCATG**GGTACC**AGTCACTGTGTTGTTTTA	F pPsaEF 5′
CPL101	CGATATC**AAGCTT**TTAACTAACGTCATGATAGGACGTATGG	R pPsaEF 3′
JQ151	CGCTCTAGAACTAGTGGATCCATTAACGGGGGCGCTGTCTATGG	F pPsaF 5′
JQ152	GCTGGGTACCGGGCCCCCCCTCGAGGTCGACATAACTCAGTCGCAGACCTATAG	R pPsaF 3′

a Restriction sites are in bold. Sequence overlaps for Gibson Assembly cloning are underlined.

b How the primer was used for cloning. The primer was used to construct the indicated plasmid or construct. F, forward primer; R, reverse primer.

### (i) *psaEF* deletion and complementation.

The plasmid for generating an in-frame deletion of *psaEF* was constructed by amplifying ∼500-bp DNA fragments upstream and downstream of *psaE* and *psaF*, respectively. These fragments were digested and cloned into pSR47S to generate pJC306. This plasmid was introduced into YP6 via conjugation, essentially as described previously (17). Briefly, transconjugants were selected on BHI plates with 50 μg/ml Kan (Kan_50_) and 2 μg/ml Irg (Irg_2_). The second recombination event was selected for by streaking colonies resistant to Kan_50_ and Irg_2_ onto BHI agar plates containing 5% sucrose. PCR was performed on candidate colonies to identify those with the deletion of *psaEF*. A single clone was selected for experimentation and named YPA18.

The plasmid used to introduce *psaE* and *psaF* at the native site on the chromosome was constructed as follows. The *psaEF* coding sequence including 500-bp upstream and downstream flanking sequences was amplified and cloned into pSR47S to generate pEW104. The plasmids expressing only *psaE* (pEW105) or only *psaF* (pEW106) were similarly constructed. For pEW105, the *psaEF* promoter region and *psaE* coding sequence were amplified. For pEW106, the *psaF* coding sequence and the *psaEF* promoter sequence were amplified separately and cloned into pSR47S. These plasmids were introduced into YPA18 (Δ*psaEF*) via conjugation and subjected to the same selection procedure used to generate deletion strains. The resulting strains are YPA260 (pEW104, *psaEF*^+^), YPA265 (pEW105, *psaE*^+^ only), and YPA279 (pEW106, *psaF*^+^ only).

### (ii) *gfp* transcriptional reporters.

To construct *gfp* transcriptional reporter plasmids, ∼500-bp fragments containing the promoter regions of *psaA* and *psaEF* were amplified and cloned into pPROBE-AT and pPROBE-gfp[tagless] (56), respectively, transformed into E. coli DH5α, and selected on LB 100 μg/ml Carb (Carb_100_) and LB Kan_50_ plates, respectively. The resulting plasmids, pEW102 (*psaA-gfp*) and pJC126 (*psaEF-gfp*), were introduced into Y. pestis strains via electroporation.

### (iii) *lacZ* translational reporters.

The Kan^r^ cassette from pKD13 (57) was amplified and cloned into pUC18-R6K-mini-Tn7T (58) to generate pEW103. Then, the *lacZ* coding sequence from pFU61 (59) was amplified and cloned into pEW103 to generate pJQ003 (see Fig. S1 in the supplemental material).

Plasmids containing the *psaE* translational fusions to *lacZ* were each made by amplifying the native *psaEF* promoter and the native or mutant *psaE* 5′ UTR (5G or 5C) and cloned into pJQ003, such that the native promoter drives the expression of the 5′ UTR and gene fusion. The *psaEF* promoter and native *psaE* 5′ UTR (no substitutions) were amplified as a single fragment that was cloned into pJQ003, generating pJQ021 (*psaE*^native^-*lacZ*). The 5C or 5G nucleotide mutations were introduced into the *psaE* 5′ UTR by first amplifying the UTR with primers containing the substituted nucleotides. These products, along with the native *psaEF* promoter fragment (up to the transcriptional start site), were then cloned into pJQ003 to generate pJQ027 (*psaE*^5C^-*lacZ*) and pJQ028 (*psaE*^5G^-*lacZ*), respectively. The *psaF* translational reporter plasmid was constructed similarly. A fragment spanning ∼800 bp upstream of the *psaF* start codon (within the *psaE* gene) was amplified and cloned along with the same native *psaEF* promoter fragment used for the *psaE* fusions into pJQ003 to generate pJQ043 (*psaF*^up^-*lacZ*). All *lacZ* reporter plasmids were electroporated into E. coli S17-1 λ*pir* and then introduced into YPA87 (Δ*lacZ*) via conjugation using a triparental mating with E. coli containing the pTNS-2 helper plasmid to mediate integration at the Tn*7* site on the chromosome (58). Transconjugates were selected on BHI plates with Kan_50_/Irg_2_ and were then patched onto BHI Kan_50_ and BHI Carb_100_ plates to identify clones that no longer contained pTNS-2.

### (iv) *psaE* 5′ UTR mutants at the native site.

A similar set of plasmids with altered *psaE* 5′ UTR sequences, as described above, was constructed for chromosomal complementation. These plasmids contain the native *psaEF* promoter and either the 5C and 5G *psaE* 5′ UTR and were amplified as described above with primers designed for Gibson Assembly into pSR47S. The resulting plasmids, pJQ029 and pJQ030, respectively, were introduced into YPA18 via conjugation and selected for growth on BHI plates with Kan_50_ and Irg_2_. The resulting complemented strains are YPA360 (*psaE*^5C^) and YPA361 (*psaE*^5G^).

### (v) *trans* complementation.

The plasmid for expressing *psaEF* from the *tet* promoter was constructed by amplifying the *psaEF* coding sequence and cloning into pMWO-005 (60). pMWO-005 contains the *tet* operator/promoter and RBS for inducible transcription and efficient translation. The resulting plasmid, pPsaEF, was introduced into YPA18 via electroporation.

The plasmid for complementation of *psaF* in *trans* was constructed as follows. The *psaEF* promoter (up to the +1 site) and a DNA fragment extending ∼800 bp upstream of *psaF* and ∼500 bp downstream were amplified, digested with SalI and BamHI, and cloned into pWKS30 (61) via Gibson Assembly. The resulting plasmid, pPsaF, was introduced into YPA265 via electroporation.

### *gfp* transcriptional reporter assay.

To analyze promoter activity, *gfp* transcriptional reporter plasmids were introduced in Y. pestis CO92 strains via electroporation. Saturated cultures were subcultured to an optical density at 600 nm (OD_600_) of 0.2 in unbuffered BHI broth or BHI broth buffered to pH 6.3, 6.7, or 7.3 and grown for 8 h with aeration at 26°C or 37°C. Relative fluorescent units (RFU) from each sample were measured using a Synergy HT microplate reader (BioTek Instruments, Winooski, VT) and normalized to the OD_600_ to determine the RFU per OD_600_ (RFU/OD_600_). For the *gfp* time course in Fig. 1A and B, strains were subcultured as described above into 30 ml unbuffered BHI broth and grown at 37°C or 26°C for 10 h with aeration. Every 2 h, a 2-ml aliquot was removed for OD_600_ and RFU measurements, and then the cells were removed by centrifugation to determine the pH of the supernatant.

### β-Galactosidase assays.

Saturated cultures of Y. pestis strains containing translational reporters grown in unbuffered BHI broth at 26°C were subcultured to an OD_600_ of 0.2 in BHI broth buffered to pH 6.3 or 7.3 and grown for 8 h at 26°C or 37°C. Assays were performed as previously described (62).

### RNA isolation and RT-PCR.

Saturated cultures of YP6 were subcultured to an OD_600_ of 0.2 in BHI broth buffered to pH 6.3 or pH 7.3 and grown at 37°C or 26°C. After 8 h, 10 OD_600_ of cells were pelleted and resuspended in 1 ml TRIzol reagent (Sigma). RNA was extracted and treated with DNase I according to the manufacturer’s (Sigma) instructions. Using 2 μg of RNA for the template, cDNA synthesis was performed with SuperScript III (Life Technologies), following the manufacturer’s protocol. RT-PCR was performed using cDNA as the template and primers for *psaE* and *psaF*. DNase I-treated RNA that was not treated with reverse transcriptase served as a control for DNA contamination, and YP6 genomic DNA (gDNA) was used as a positive PCR control. RT-PCR products were separated on a 1% agarose gel and visualized by staining with GelRed nucleic acid stain (Biotium).

### Peptide synthesis and antibody production.

To generate antibodies that recognize PsaE and PsaF, two PsaE and PsaF peptide fragments containing keyhole limpet hemocyanin (KLH) conjugations were synthesized by LifeTein (Somerset, NJ). The synthesized peptides were sent to Cocalico Biologicals, Inc. (Stevens, PA), and both peptide fragments were used for immunization of New Zealand White rabbits, following standard protocols. To generate an antibody against PsaA, a His-PsaA fusion protein was expressed in E. coli, purified, and sent to Covance Research Products, Inc. (Denver, PA) for immunization of a New Zealand White rabbit, following standard protocols.

### Western blot analysis.

Saturated cultures of indicated strains were subcultured to an OD_600_ of 0.2 in BHI broth buffered to pH 6.3 or 7.3 and grown for 8 h at 26°C or 37°C. Whole-cell lysates were prepared from 1.5 ml of cells that were pelleted, washed once with ice-cold phosphate-buffered saline (PBS), and resuspended in Laemmli buffer containing 5% β-mercaptoethanol. Samples were boiled for 10 min, and 0.2 OD_600_ separated via SDS-PAGE and transferred to polyvinylidene difluoride (PVDF) membrane for Western blot analysis. Loading was qualitatively assessed by Ponceau S staining of the PVDF membrane. Anti-PsaE, anti-PsaF, and anti-PsaA sera were used to probe for PsaE, PsaF, and PsaA, respectively. Prior to use, the anti-PsaE serum was adsorbed against Y. pestis Δ*psaEF* mutant lysates and was used at a titer of 1:100. Anti-PsaF serum was used at a titer of 1:1,000. Anti-PsaA serum was absorbed against E. coli lysates and used at a titer of 1:2,500. Anti-IgG horseradish peroxidase (HRP)-conjugated secondary antibodies were used at a titer of 1:20,000.

### Statistical analysis.

Analyses were performed using GraphPad Prism v8.0 (San Diego, CA).

## Supplementary Material

Supplemental file 1
