# Supplementary material for: Temperature Control of psaA Expression by PsaE and PsaF in Yersinia pestis
Source: J Bacteriol. 2019 Jul 24;201(16):e00217-19. doi: 10.1128/JB.00217-19 (PMC6657601; doi:10.1128/JB.00217-19)
Supplement: Supplemental file 1 [file JB.00217-19-s0001.pdf]

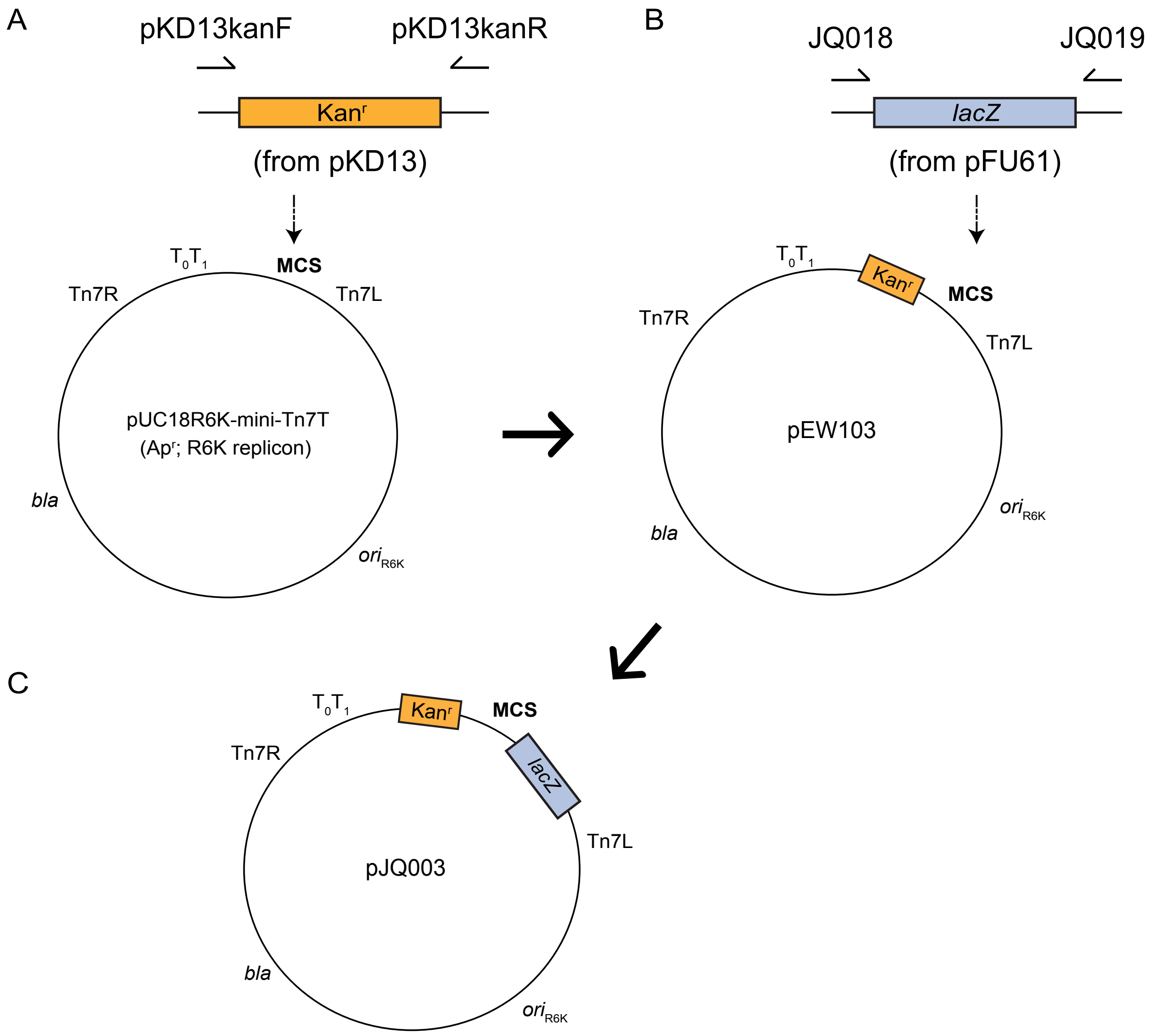

Supplemental Fig 1. Map depicting construction of *lacZ* translational reporter plasmid pJQ003 (A-C). (A) The Kan^r cassette from pKD13 was amplified using the primers indicated and cloned into the EcoRV site of pUC18R6K-mini-Tn7T to generate pEW103. (B) The *lacZ* sequence from pFU61 was amplified using the primers indicated and cloned into pEW103 digested with XhoI/KpnI to generate pJQ003 (C). pJQ003 contains a promoterless *lacZ* downstream of the MCS. A ribosome-binding site (RBS) must be included to ensure translation of *lacZ*. This vector includes a R6K replication origin (ori_{R6K}), a β-lactamase encoding gene (*bla*), transcriptional terminators (T_0, T_1), and the left and right end of Tn7 (Tn7L and Tn7R, respectively).
